# The observational constraints on the flat $\phi$CDM models

**DOI:** 10.1140/epjc/s10052-018-6233-y

**Published:** 2018-09-26

**Authors:** Olga Avsajanishvili, Yiwen Huang, Lado Samushia, Tina Kahniashvili

**Affiliations:** 1 Abastumani Astrophysical Observatory, Ilia State University, 3-5 Cholokashvili Ave., 0194 Tbilisi, Georgia; 2 Department of Physics, University of California, San Diego, La Jolla, CA 92093 USA; 3 Department of Physics, Kansas State University, 116 Cardwell Hall, Manhattan, KS 66506 USA; 4 McWilliams Center for Cosmology and Department of Physics, Carnegie Mellon University, 5000 Forbes Ave, Pittsburgh, PA 15213 USA; 5 Department of Physics, Laurentian University, Ramsey Lake Road, Sudbury, ON P3E 2C Canada

## Abstract

Most dark energy models have the $\varLambda$CDM as their limit, and if future observations constrain our universe to be close to $\varLambda$CDM Bayesian arguments about the evidence and the fine-tuning will have to be employed to discriminate between the models. Assuming a baseline $\varLambda$CDM model we investigate a number of quintessence and phantom dark energy models, and we study how they would perform when compared to observational data, such as the expansion rate, the angular distance, and the growth rate measurements, from the upcoming Dark Energy Spectroscopic Instrument (DESI) survey. We sample posterior likelihood surfaces of these dark energy models with Monte Carlo Markov Chains while using central values consistent with the Planck $\varLambda$CDM universe and covariance matrices estimated with Fisher information matrix techniques. We find that for this setup the Bayes factor provides a substantial evidence in favor of the $\varLambda$CDM model over most of the alternatives. We also investigated how well the CPL parametrization approximates various scalar field dark energy models, and identified the location for each dark energy model in the CPL parameter space.

## Introduction

It is well established that our universe is undergoing an accelerating expansion today [1–3]. Several observations suggest that this accelerated expansion started relatively recently at $z\sim 0.7$ [4, 5]. One of the possible explanations is to assume the presence of dark energy as a dominant component of the total energy density budget in the universe today (i.e. around 70% of the universe matter–energy content today is a substance with negative pressure that drives today’s accelerated expansion). Dark energy is characterized by an equation of state (EOS) parameter *w* defined by as a ratio between the pressure (*p*) and the energy density ($\rho$), $w \equiv p/\rho$. The accelerated expansion requires that $w <-1/3$. Generally speaking *w* parameter might be time dependent. In the framework of the standard cosmological (concordance) model, dark energy is represented by the cosmological constant $\varLambda$ (that was originally introduced by Albert Einstein, and it is assumed to be associated with the vacuum energy density). This cosmological model is referred as $\varLambda$CDM model, in this case the EOS parameter is constant, $w=-1$. The $\varLambda$CDM model is simple and easy to constrain through observations, but besides good agreements with existing observational data, the model has a number of shortcomings (the cosmological constant problem, the coincidence problem, the matter – anti-matter asymmetry, the weakness of gravity compared to other forces, etc.) [6–10]. The most notable of these puzzles is the cosmological constant problem which stems from the fact that the theoretically expected value (based on quantum field theory approach and on dimensional arguments) of the cosmological constant associated energy density is determined by $M_\mathrm{pl}^4$ (where $M_\mathrm{pl}=1.2211 \times 10^{19}$ GeV is the Planck mass), while the actual value (suggested through observational data) is order of 120 magnitudes lower [11–13]. In order to overcome this (and other) difficulties (the coincidence problem, for example), *dynamical* dark energy models were proposed [14, 15], and see Ref. [16] for a recent review.

Several large scale structure surveys missions, such as e.g. Dark Energy Spectroscopic Instrument (DESI), Wide-Field Infrared Survey Telescope (WFIRST) and Euclid are scheduled to start operating within the next decade. Upon completion of these missions, very accurate measurements of the expansion velocity, angular distance and growth rate in the universe to redshifts of $z\sim 2$ will be obtained [17–21]. These measurements cumulatively have a very strong constraining power on the behavior of both dark energy and gravity on large length scales. If the $\varLambda$CDM model is *not* the correct cosmological model, we should be able to see this in upcoming data. If, however, the $\varLambda$CDM model or a model very close to it, is the correct model, the interpretation of the data will be less straightforward. One reason for this is that the most viable dark energy models have the $\varLambda$CDM model as their limit so the Bayesian arguments about the fine-tuning of the extra parameters will have to be employed. In this work we refer to a *simulated* DESI data and study how these models would perform when compared to the baseline $\varLambda$CDM model. The main question we ask is if the $\varLambda$CDM were the correct model of cosmology would we be able to unambiguously discard alternative models based on DESI data.

In this paper we investigate a representative family of dark energy models that are based on the idea of a cosmological scalar field [22–26]. If the scalar field, $\phi$, has a slowly rolling stage, the energy density associated with this field can mimic the presence of the cosmological constant at late stages. There are many proposals for the functional form of the self-interacting potential of the scalar field that are allowed by the current observational data [27–40]. In this paper we consider two types models: the quintessence (dark energy is presented in the form of a canonical scalar fields) and the phantom models (dark energy is presented in the form of a non-canonical scalar field). As of now, there is no consensus on which of these models is preferable based on the results obtained from the different observations [41–45]. We study the scalar field models with 10 quintessence and 7 phantom potentials in the Bayesian framework [46–48]. We also limit ourselves by considering the flat scalar field dark energy (so called $\phi$CDM) models. This is justified by the fact that large deviations from the spatial flatness of the universe seem to be well constrained by the CMB data [44]. We have found that under these assumptions a vast majority of the scalar field dark energy models will be characterized by low enough Bayes factors to suggest a substantial preference for the $\varLambda$CDM model.

The paper is organized as follows: in Sect. 2 we review the dark energy models (including the scalar field quintessence and phantom models); in Sect. 3 we describe observational tests, our results are presented in Sec. 4, and we conclude in Sect. 5. We use natural units: $c=1=k_B=1$ throughout the paper.

## Dark energy models

We will consider two families of scalar field dark energy (flat) models: the quintessence (canonical) and the phantom scalar field (non-canonical) models. These models have opposite properties in their manifestation today: (1) in the range of the EOS parameter values ($w<-1$ for the phantom field and $-1/3<w<-1$ for the quintessence field); (2) in the sign of the kinetic term in the Lagrangian (the negative sign for the phantom field and the positive one for the quintessence field); (3) in the dynamics of the scalar fields (the quintessence field rolls to the minimum its potential, the phantom field rolls to the “uphill” its potential); (4) in the dynamics of the dark energy density (increases over time for the phantom field and almost doesn’t change over time for the quintessence field); (5) in the forecast for the future evolution of the universe: for the phantom models violent future events (such as big/little/pseudo rips) are predicted, while in the quintessence models either an eternal expansion or a re-collapse depending on the spatial curvature of the universe is predicted.

The action associated with the scalar field, $\phi$, is given field by [49]:

1 $$\begin{aligned} S=\frac{M_\mathrm{pl}^2}{16\pi } \int {d^{4}x\Bigl [\sqrt{-g} \Bigl (\pm \frac{1}{2}g^{\mu \nu }\partial _\mu \phi \partial _\nu \phi - V(\phi )\Bigr )\Bigr ]}, \end{aligned}$$

where “$+$” sign before the kinetic term ($g^{\mu \nu }\partial _\mu \phi \partial _\nu \phi /2$) refers to the quintessence models, while “−” stands for the phantom models; $g^{\mu \nu }$ is the background metric,^1^ and $V(\phi )$ is the self-interacting potential of the scalar field, $\phi$. The scalar field is assumed to exhibit the negligible spatial variations, so that the spatial derivatives are small compared to the time derivatives, and thus we assume the scalar field to be an homogeneous field.

Varying the action Eq. (1), the Klein–Gordon scalar field equation of motion can be obtained [50]:

2 $$\begin{aligned} \ddot{\phi }+3\frac{\dot{a}}{a}\dot{\phi }\pm \frac{\partial V(\phi )}{\partial \phi }=0, \end{aligned}$$

where again “$+/-$” sign corresponds to the quintessence/

phantom model respectively, the over-dot denotes a derivative with respect to the physical time, *t*.

The energy density and the pressure of the scalar field are expressed [51]:

3 $$\begin{aligned} \rho _\phi = \frac{M_\mathrm{pl}^2}{32\pi } \Bigl (\pm \dot{\phi }^2/2 + V(\phi ) \Bigr ), \end{aligned}$$

4 $$\begin{aligned} P_\phi = \frac{M_\mathrm{pl}^2}{32\pi } \Bigl (\pm \dot{\phi }^2/2 - V(\phi ) \Bigr ), \end{aligned}$$

and the effective EOS parameter for the scalar field is then given by $w_\phi = \dfrac{\pm {\dot{\phi }}^2/2 - V(\phi )}{{\pm \dot{\phi }}^2/2 + V(\phi )}$. If the time-derivatives of the scalar field, $\phi$, are small enough to make the magnitude of the kinetic term small compared to the potential $|\pm {\dot{\phi }}^2/2| \ll V(\phi )$ (the “slow roll” condition [22]), the EOS parameter is very close to negative one^2^ and the scalar field behaves like a slowly-time-varying cosmological constant (sometimes the scalar field is referred as a slowly rolling scalar field Ref. [10]). Below we list the potentials of the quintessential and phantom models considered in this work:

### The quintessence models

- Ratra–Peebles potential: $V(\phi )=V_0M_\mathrm {pl}^2\phi ^{-\alpha }$; $\alpha =\mathrm{const} >0$ [23]
- Ferreira–Joyce potential:^3^ $V(\phi )=V_0\exp (-\lambda \phi /M_\mathrm{pl})$; $\lambda = \mathrm{const}>0$ [27]
- Zlatev–Wang–Steinhardt potential: $V(\phi )=V_0(\exp ({M_\mathrm{pl}/\phi })-1)$ [28]
- Sugra potential: $$V(\phi )=V_0\phi ^{-\chi }\exp (\gamma \phi ^2/M_\mathrm{pl}^2)$$; $$\chi , \gamma =\mathrm{const}>0$$ [29]
- Sahni–Wang potential: $V(\phi )=V_0(\cosh (\varsigma \phi )-1)^g$; $\varsigma =\mathrm{const}>0$, $g=\mathrm{const}<1/2$ [30]
- Barreiro–Copeland–Nunes potential: $V(\phi )=V_0(\exp (\nu \phi ) + \exp (\upsilon \phi ))$; $\nu$, $\upsilon =\mathrm{const}\ge 0$ [31]
- Albrecht–Skordis potential: $V(\phi )=V_0((\phi -B)^2 +A)\exp (-\mu \phi )$; *A*, $B=\mathrm{const}\ge 0$, $\mu =\mathrm{const}>0$ [32]
- Urẽna–López–Matos potential: $V(\phi )=V_0\sinh ^m(\xi M_\mathrm {pl}\phi )$; $\xi =\mathrm{const}>0$,$m=\mathrm{const}<0$ [33]
- Inverse exponent potential: $V(\phi )=V_0\exp ({M_\mathrm{pl}/\phi })$ [34]
- Chang–Scherrer potential: $V(\phi )=V_0(1+\exp (-\tau \phi ))$;$\tau =\mathrm{const}>0$ [35]

### The phantom models

- Fifth power potential: $V(\phi )=V_0\phi ^5$ [36]
- Inverse square potential: $V(\phi )=V_0\phi ^{-2}$ [36]
- Exponent potential: $$V(\phi )=V_0\exp (\beta \phi )$$; $$\beta = \mathrm{const} >0$$ [36]
- Quadratic potential: $$V(\phi )=V_0\phi ^2$$ [37]
- Gaussian potential: $$V(\phi )=V_0(1-\exp (\phi ^2/\sigma ^2))$$; $$\sigma =\mathrm{const}$$ [37]
- Pseudo Nambu-Goldstone boson potential: $V(\phi )=V_0(1-\cos (\phi /\kappa ))$; $\kappa = \mathrm{const} >0$ [38]
- Inverse hyperbolic cosine potential: $V(\phi )=V_0(\cosh (\psi \phi ))^{-1}$; $\psi = \mathrm{const} >0$ [39]

In both cases of the quintessence and the phantom models, $V_0$ is the model parameter with the dimension of GeV$^4$. This parameter is obviously related to the dark energy density parameter today.

## Testing dark energy potentials

### Model description

To see how well we will be able to discriminate between these dark energy scalar field potentials after upcoming dark energy surveys, we generate a set of the simulated data (theoretical model predictions) for the Hubble expansion rate, the angular distance, and the growth rate, in the redshift range of $0.15<z<1.85$ (with $z=1/a - 1$ is the redshift) expected from DESI mission [17]. The measurements are centered around their true values in our fiducial cosmology with the errorbars based on the Fisher matrix predictions. We compute the theoretical expectation for the angular distance, Hubble parameter, and the growth rate and treat them as measurements for our mock data set. We use the standard Fisher matrix predictions for the covariance of these measurements. The real DESI data will, of course, be a random realization from the likelihood space that doesn’t necessarily sit on top of the maximum likelihood, and the Fisher matrix predictions tend to overestimate the constraining power of the data. We don’t expect these effects big enough to significantly affect our conclusion. We fit this synthetic data by using the standard MCMC analysis method to estimate the multidimensional posterior likelihood of the model parameters.

For all dark energy models we compute:

1. *The Hubble parameter H(z)*: The first Friedmann equation for the flat universe is [10]:
  5 $$\begin{aligned} E^2(z) = \varOmega _\mathrm{r,0}(1+z)^{4}+\varOmega _\mathrm{m,0}(1+z)^{3}+ \varOmega _\mathrm{\phi }(z), \end{aligned}$$
  here $E(z) = H(z)/H_{\mathrm {0}}$ is the normalized Hubble parameter, and $H_{\mathrm {0}}$ is the Hubble parameter today; $\varOmega _{i}(z) \equiv \rho _i(z)/\rho _\mathrm{cr}$ is the energy density parameter for “i”-th component (characterized by the energy density, $\rho _i(z)$).^4^
2. *The angular diameter distance* Assuming a flat universe, the angular diameter distance is given by [49]:
  6 $$\begin{aligned} d_A(z)=\frac{1}{H_{\mathrm {0}}(1+z)}\int _0^z\frac{dz'}{E(z')} \end{aligned}$$
3. *The combination of the growth rate and the matter power spectrum amplitude,* $f(a)\sigma _8(a)$

The growth rate is given as, $f(a)={\mathrm {d}}\mathrm {ln}D(a)/{\mathrm {d}}\mathrm {ln}a$, where *D*(*a*) is the growth function defined through the ratio of overdensities, $\delta (a)$, at different scale factors, as $D(a)=\delta (a)/\delta (a_0)$, normalized to be unity today, ($D(a_0)=1$), and it is a solution of the following linear perturbation equation [53]:

7 $$\begin{aligned} D^{''}+\Bigl (\frac{3}{a}+\frac{E^{'}}{E}\Bigr )D^{'} -\frac{3\varOmega _{\mathrm {m,0}}}{2a^{5}E^{2}}D=0, \end{aligned}$$

here a prime denotes a derivative with respect to the scale factor, *a*, ($^\prime = d/da$). The matter power spectrum amplitude can be characterized through the $\sigma _8(a)$ function, $\sigma _8(a)\equiv D(a)\sigma _8$, where $\sigma _8 \equiv \sigma _8(a_0)$ is the rms linear fluctuation in the mass distribution on scales $8h^{-1}$ Mpc (with *h* is the today Hubble constant in units of 100 km / s/Mpc) today. We fix the value of $\sigma _8$ to its current best-fit $\varLambda CDM$ value of $\sigma _8=0.815$ from the Plank 2015 data [44], (see Ref. [54] for model-independent cosmological constraints on $\sigma _8$ from growth and expansion).

The EOS parameter of the dark energy models is often characterized by the Chevallier–Polarsky–Linder (CPL)

**Table 1:** The list of the dark energy quintessence potentials and the free parameters

The quintessence potentials	Free parameters	
$V(\phi )=V_0M_\mathrm {pl}^2\phi ^{-\alpha }$	$H_\mathrm {0}$ (50–90)	$V_0$ (3–5)
	$\varOmega _\mathrm {m0}$ (0.25–0.32)	$\alpha$ (10$^{-6}$–0.7)
$V(\phi )=V_0\exp (-\lambda \phi /M_\mathrm{pl})$	$H_\mathrm {0}$ (50–90)	$\lambda \,(10^{-7}{-}10^{-3})$
	$\varOmega _\mathrm {m0}\,(0.25{-}0.32)$	$\phi _0\,(0.2{-}1.6)$
	$V_0\,(10{-}10^3)$	$\dot{\phi _0}\,(79.8{-}338.9)$
$V(\phi )=V_0(\exp ({M_\mathrm{pl}/\phi })-1)$	$H_\mathrm {0}\,(50{-}90)$	
	$\varOmega _\mathrm {m0}\,(0.25{-}0.32)$	$\phi _0\,(1.5{-}10)$
	$V_0\,(10{-}10^2)$	$\dot{\phi _0}\,(350{-}850)$
$V(\phi )=V_0\phi ^{-\chi }\exp (\gamma \phi ^2/M_\mathrm{pl}^2)$	$H_\mathrm {0}\,(50{-}90)$	
	$\varOmega _\mathrm {m0}\,(0.25{-}0.32)$	$\gamma \,(6.5{-}7)$
	$V_0\,(10^{-2}{-}10^{-1})$	$\phi _0\,(5.78{-}10.55)$
	$\chi \,(4{-}8)$	$\dot{\phi _0}\,(680.6{-}879)$
$V(\phi )=V_0(\cosh (\varsigma \phi )-1)^g$	$H_\mathrm {0}\,(50{-}90)$	
	$\varOmega _\mathrm {m0}\,(0.25{-}0.32)$	$g\,(0.1{-}0.49)$
	$V_0\,(5{-}8)$	$\dot{\phi _0}\,(360{-}685)$
	$3\varsigma \,(0.15{-}1)$	$\phi _0\,(1.8{-}5.8)$
$V(\phi )=V_0(\exp (\nu \phi ) + \exp (\upsilon \phi ))$	$H_\mathrm {0}\,(50{-}90)$	$\nu \,(6{-}12)$
	$\varOmega _\mathrm {m0}\,(0.25{-}0.32)$	$\phi _0\,(0.014{-}1.4)$
	$V_0\,(1{-}12)$	$\dot{\phi _0}\,(9.4{-}311)$
$V(\phi )=V_0((\phi -B)^2 + A)\exp (-\mu \phi )$	$H_\mathrm {0}\,(50{-}90)$	$B\,(1{-}60)$
	$\varOmega _\mathrm {m0}\,(0.25{-}0.32)$	$\mu \,(0.2{-}0.9)$
	$V_0\,(40{-}70)$	$\phi _0\,(5.8{-}8.45)$
	$A\,(1{-}40)$	$\dot{\phi _0}\,(681{-}804.5)$
$V(\phi )=V_0\sinh ^m(\xi M_\mathrm {pl}\phi )$	$H_\mathrm {0}\,(50{-}90)$	
	$\varOmega _\mathrm {m0}\,(0.25{-}0.32)$	$\xi \,(10^{-2}{-}1)$
	$V_50\,(1{-}10)$	$\phi _0\,(0.5{-}2.5)$
	$m\,(-0.1{-}-0.3)$	${{\dot{\phi }}_0}\,(190{-}367)$
$V(\phi )=V_0\exp ({M_\mathrm{pl}/\phi })$	$H_\mathrm {0}\,(50{-}90)$	
	$\varOmega _\mathrm {m0}\,(0.25{-}0.32)$	$\phi _0\,(5.78{-}10.55)$
	$V_0\,(10^2{-}10^3)$	$\dot{\phi _0}\,(680.6{-}879)$
$V(\phi )=V_0(1+\exp (-\tau \phi ))$	$H_\mathrm {0}\,(50{-}90)$	$\tau \,(10{-}10^2)$
	$\varOmega _\mathrm {m0}\,(0.25{-}0.32)$	$\phi _0\,(0.01{-}0.075)$
	$V_0\,(1{-}10^2)$	$\dot{\phi _0}\,(9.4{-}32)$

**Table 2:** The list of the dark energy phantom potentials and the free parameters

The phantom potentials	Free parameters	
$V(\phi )=V_0\phi ^5$	$H_\mathrm {0}\,(50{-}90)$	
	$\varOmega _\mathrm {m0}\,(0.25{-}0.32)$	$\phi _0\,(3.37{-}3.94)$
	$V_0\,(10^{-3}{-}10^{-2})$	$\dot{\phi _0}\,(523{-}563.6)$
$V(\phi )=V_0\phi ^{-2}$	$H_\mathrm {0}\,(50{-}90)$	
	$\varOmega _\mathrm {m0}\,(0.25{-}0.32)$	$\phi _0\,(2.83{-}5.15)$
	$V_0\,(30{-}50)$	$\dot{\phi _0}\,(471.4{-}600)$
$V(\phi )=V_0\exp (\beta \phi )$	$H_\mathrm {0}\,(50{-}90)$	$\beta \,(0.08{-}0.3)$
	$\varOmega _\mathrm {m0}\,(0.25{-}0.32)$	$\phi _0\,(0.2{-}9.14)$
	$V_0\,(1{-}20)$	$\dot{\phi _0}\,(79.8{-}830.9)$
$V(\phi )=V_0\phi ^2$	$H_\mathrm {0}\,(50{-}90)$	
	$\varOmega _\mathrm {m0}\,(0.25{-}0.32)$	$\phi _0\,(0.67{-}2.8)$
	$V_0\,(1{-}20)$	$\dot{\phi _0}\,(191{-}450)$
$V(\phi )=V_0(1-\exp (\phi ^2/\sigma ^2))$	$H_\mathrm {0}\,(50{-}90)$	$V_0\,(5{-}30)$
	$\varOmega _\mathrm {m0}\,(0.25{-}0.32)$	$\phi _0\,(0.67{-}2.8)$
	$\sigma \,(5{-}30)$	$\dot{\phi _0}\,(191{-}450)$
$V(\phi )=V_0(1-\cos (\phi /\kappa ))$	$H_\mathrm {0}\,(50{-}90)$	$\kappa \,(1.1{-}2)$
	$\varOmega _\mathrm {m0} \,(0.25{-}0.32)$	$\phi _0\,(2.3{-}3.37)$
	$V_0\,(1{-}4)$	$\dot{\phi _0}\,(420{-}500)$
$V(\phi )=V_0(\cosh (\psi \phi ))^{-1}$	$H_\mathrm {0}\,(50{-}90)$	$\psi \,(10^{-3}{-}1)$
	$\varOmega _\mathrm {m0}\,(0.25{-}0.32)$	$\phi _0\,(1.4{-}2.3)$
	$V_0\,(10^{-3}{-}10^2)$	$\dot{\phi _0}\,(310{-}420.7)$

$$w_0-w_a$$ parametrization [55, 56]:

8 $$\begin{aligned} w(a) = w_0 + w_a(1-a), \end{aligned}$$

where $$w_0=w(a=1)$$ and $$w_a=-a^{-2}(\mathrm{d} w/\mathrm{d}a)|_{a=1/2}$$.

This parametrization fits the EOS parameters for most of the dark energy models well enough for some effective values of $w_0$ and $w_a$, but may fail to describe the arbitrary dark energy models to a good precision (few percents) over a wide redshift range.^5^

In addition, the structure growth (in the most dark energy models) tends to be sensitive (only) to the fractional matter density, $\varOmega _\mathrm{m}(a) = \varOmega _\mathrm{m}/E^2(a)$, with $\varOmega _\mathrm{m}=\varOmega _\mathrm{m,0}a^{-3}$ and as a consequence, the matter perturbation growth rate function, *f*(*a*), with high accuracy can be parameterized as [58]:

9 $$\begin{aligned} f(a) \approx [\varOmega _\mathrm{m}(a)]^{\gamma (a)}, \end{aligned}$$

where $\gamma (a)$ is so called *the growth index*, and in general it is a time-dependent function.^6^ In the case of the *w*CDM models (or any dark energy models which are the well approximated by the $w_0-w_a$ parametrization), the growth index, $\gamma (a)$, scale factor dependence on can be determined from Eq. (9), see Ref. [61]:

10 $$\begin{aligned} \gamma (a) = \frac{\ln f(a)}{\ln \varOmega _\mathrm{m}(a)}. \end{aligned}$$

On the other hand, the function, $\gamma (a)$, can be parameterized by a scale factor independent manner, so called the Linder $\gamma$-parametrization, see Ref. [63]:

11 $$\begin{aligned} \gamma =\left\{ \begin{array}{rl} 0.55+0.05(1+w_0+0.5w_a),&{} \text{ if } w_0 \ge -1;\\ 0.55+0.02(1+w_0+0.5w_a),&{} \text{ if } w_0<-1. \end{array}\right. \ \end{aligned}$$

This parametrization is accurate up to redshift of $z=5$ ($a=0.2$) [60]. The numerical value of the $\gamma$ itself depends on the dark energy model characteristics (*w*-parameter), being equal to 0.55 for the $\varLambda$CDM model [63].

We don’t use the Linder $\gamma$-parametrization or CPL one in our MCMC chains. Instead we fit directly to the model predictions by solving the fundamental differential equations. We do however, as an independent exercise, check how well these parametrizations work for the dark energy models that we consider. We find that all dark energy models under consideration can be approximated very well by these two parametrizations.

### The definition of the starting points for the MCMC chains

To find the starting points for our MCMC chains, we solve jointly the scalar field equation for the quintessence and phantom models, Eq. (2), the Friedmann equation, Eq. (5), and the linear perturbation equation, Eq. (7), for a wide range of the free parameters and the initial conditions for matter dominated epoch. For each potential we have found the plausible solutions, for which the following three criteria were simultaneously fulfilled:

1. The transition between the matter and dark energy equality ($\varOmega _\mathrm {m}=\varOmega _\mathrm {\phi }$) happens relatively recently $z\in (0.6 - 0.8)$ [64].
2. The matter perturbation growth rate, *f*(*a*), and the fractional matter density, $\varOmega _\mathrm{m}(a)$, are parameterized by the Linder $\gamma$-parametrization (Eq. (11)).
3. The EOS parameter predicted by the different dark energy models should be in the agreement with the expected EOS parameter value today (for the phantom models $w_0<-1$; for the quintessence models with $-1<w_0<-0.75$: for the freezing type $w_a<0$ and for the thawing type $w_a>0$).

For all potentials we found the range for (1) the allowed initial conditions and (2) the model parameters, which we then used as the starting points for the MCMC chains.

This is done to make sure that the MCMC chains converge faster by starting them close to the peak of the posterior likelihood. The actual likelihood surface from the converged MCMC chains of course doesn’t depend on the starting point.

## Results

We computed the projected covariance matrix of $D_A(z)$, *H*(*z*), and $f\sigma _8(z)$ measurements following standard Fisher matrix approach described in Ref. [17]. We assumed 14,000 sq. deg. of sky coverage and wavenumbers up to $k_\mathrm {max} = 0.2\ \mathrm {Mpc}/h$. Our variances matched the numbers in Table V of [17]. We also accounted for covariances between the measurements within the same redshift bin. $D_A(z)$ and *H*(*z*) measurements are negatively correlated by approximately 40%, while correlations with $f\sigma _8(z)$ are below 10% for all redshift bins.

All dark energy models considered in this work have the following free parameters, $\varOmega _{\mathrm {m,0}}$ and $H_{\mathrm {0}}$. In addition, the scalar field models have the extra parameters describing the strength and shape of the potential, $V(\phi )$. These free parameters along with the prior ranges considered in our MCMC runs are presented in the Tables 1 and 2. We have found these priors using the phenomenological method, which is described in the previous section, i.e. they correspond to the three conditions imposed on the solutions for each potential. We have explicitly checked that most of the high likelihood regions are inside these priors in a way that the parameter constraints will not be effected by adjusting the prior ranges.

**Table 3:** The list of the dark energy quintessence potentials, with corresponding *AIC*, *BIC*, and Bayes factors

Quintessence potentials	AIC	BIC	Bayes factor
$V(\phi )=V_0M_\mathrm {pl}^2\phi ^{-\alpha }$	10	18.7	0.5293
$V(\phi )=V_0\exp (-\lambda \phi /M_\mathrm{pl})$	12	22.4	0.0059
$V(\phi )=V_0(\exp ({M_\mathrm{pl}/\phi })-1)$	10	18.7	0.0067
$V(\phi )=V_0\phi ^{-\chi }\exp (\gamma \phi ^2/M_\mathrm{pl}^2)$	14	26.2	0.0016
$V(\phi )=V_0(\cosh (\varsigma \phi )-1)^g$	14	26.2	0.0012
$V(\phi )=V_0(\exp (\nu \phi ) + \exp (\upsilon \phi ))$	14	26.2	0.0053
$V(\phi )=V_0((\phi -B)^2 + A)\exp (-\mu \phi )$	16	29.9	0.0034
$V(\phi )=V_0\sinh ^m(\xi M_\mathrm {pl}\phi )$	14	26.2	0.0014
$V(\phi )=V_0\exp ({M_\mathrm{pl}/\phi })$	10	18.7	0.0077
$V(\phi )=V_0(1+\exp (-\tau \phi ))$	12	22.4	0.0024

**Table 4:** The list of the dark energy phantom potentials, with corresponding *AIC*, *BIC*, and Bayes factor values

Phantom potentials	AIC	BIC	Bayes factor
$V(\phi )=V_0\phi ^5$	10	18.7	0.0921
$V(\phi )=V_0\phi ^{-2}$	10	18.7	0.0142
$V(\phi )=V_0\exp (\beta \phi )$	22.4	12	0.0024
$V(\phi )=V_0\phi ^2$	10	18.7	0.0808
$V(\phi )=V_0(1-\exp (\phi ^2/\sigma ^2))$	12	22.4	0.0113
$V(\phi )=V_0(1-\cos (\phi /\kappa ))$	12	22.4	0.0061
$V(\phi )=V_0(\cosh (\psi \phi ))^{-1}$	12	22.4	0.0056

## The Bayesian statistics

The reconstruction (and constraining) of the dark energy potentials with minimal priors is a challenging task (see e.g. [65] for more details). To assess the quality of the different models and to distinguish them from each other, we have applied the Akaike information criterion (*AIC*) [66] and the Bayesian (or Schwarz) information criterion (*BIC*) [67]. The information obtained by these criteria complement each other.

*AIC* and *BIC* are defined respectively as,

12 $$\begin{aligned} AIC = -2\ln {{\mathcal {L}}_{max}} + 2k, \end{aligned}$$

and

13 $$\begin{aligned} BIC = -2\ln {{\mathcal {L}}_{max}} + k\ln {N}, \end{aligned}$$

where ${\mathcal {L}}_{max}\propto \mathrm {exp}(-\chi ^2_\mathrm {min}/2)$ is a maximum value of the likelihood function; *N* is a number of free parameters; *k* is a number of data points.

We also computed the evidence integral defined as,

14 $$\begin{aligned} {\mathcal {E}} = \int {\mathrm {d}}^3\varvec{p} {\mathcal {P}}(\varvec{p}), \end{aligned}$$

where $\varvec{p}$ are all parameters of the model, ${\mathcal {P}}$ is the posterior likelihood (proportional to the local density of MCMC points), and the boundaries of the integral are given by the prior. We explored how tight the prior on the extra parameters needs to be for them to be competitive (in the sense of the Bayesian evidence) with the standard $\varLambda$ CDM model.

We explicitly checked that the priors incorporate most of the high posterior area. Since all dark energy models have the $\varLambda$ CDM model as their limit, ruling them out simply based on the posterior is technically speaking impossible. Since the synthetic data was generated in the $\varLambda$ CDM model that limit will always result in high likelihood, and because of the finite size of the errorbars there will always be a region around the best-fit $\varLambda$ CDM model that is consistent with the data. One could however appeal to the Bayesian evidence and argue that the extra parameters need to be extremely fine tuned. We numerically integrated the posterior likelihood to get for all models.

**Fig. 1:**
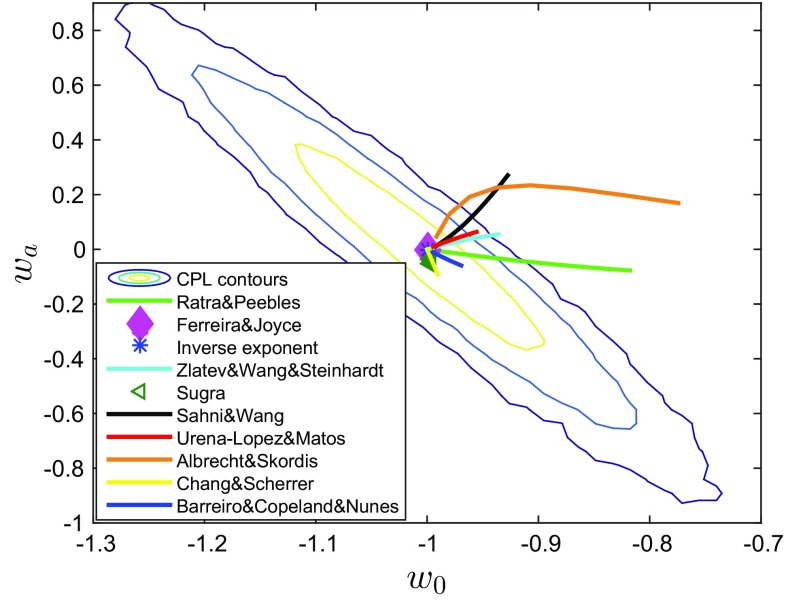
The comparison of the possible $(w_0,w_a)$ values of the quintessence dark energy potentials with the CPL-$\varLambda$CDM 3$\sigma$ confidence level contours

**Fig. 2:**
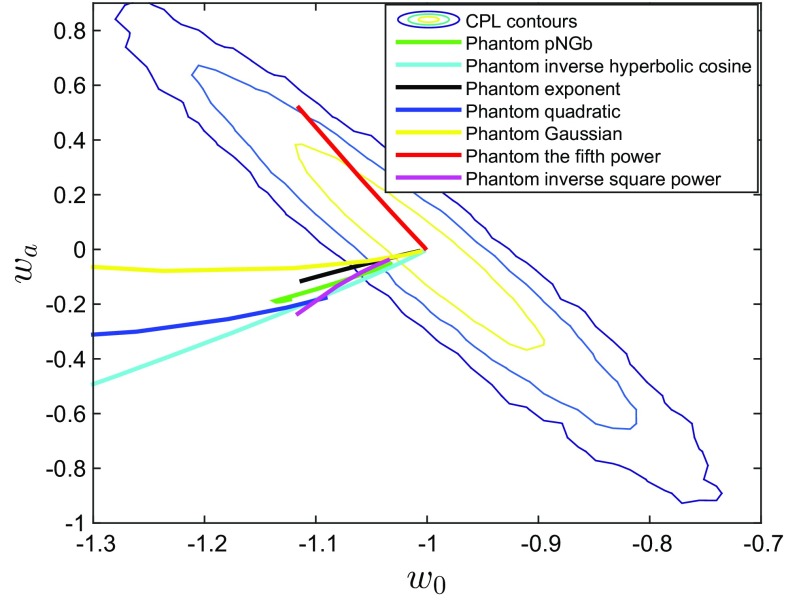
Similar to the Fig. 1, for the phantom models

**Fig. 3:**
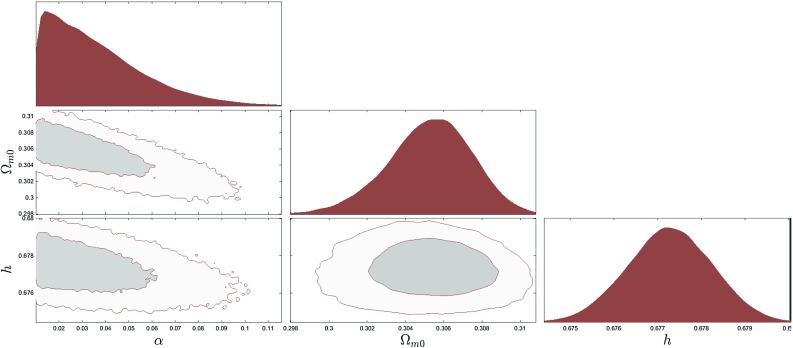
The 2$\sigma$ confidence level contour plots for various pairs of the free parameters ($\alpha$, $\varOmega _\mathrm{m0}$, *h*) for which the $\phi$CDM model with the Ratra-Peebles potential $V(\phi )=V_0M_\mathrm {pl}^2\phi ^{-\alpha }$ is in the best fit with the $\varLambda$CDM model

**Fig. 4:**
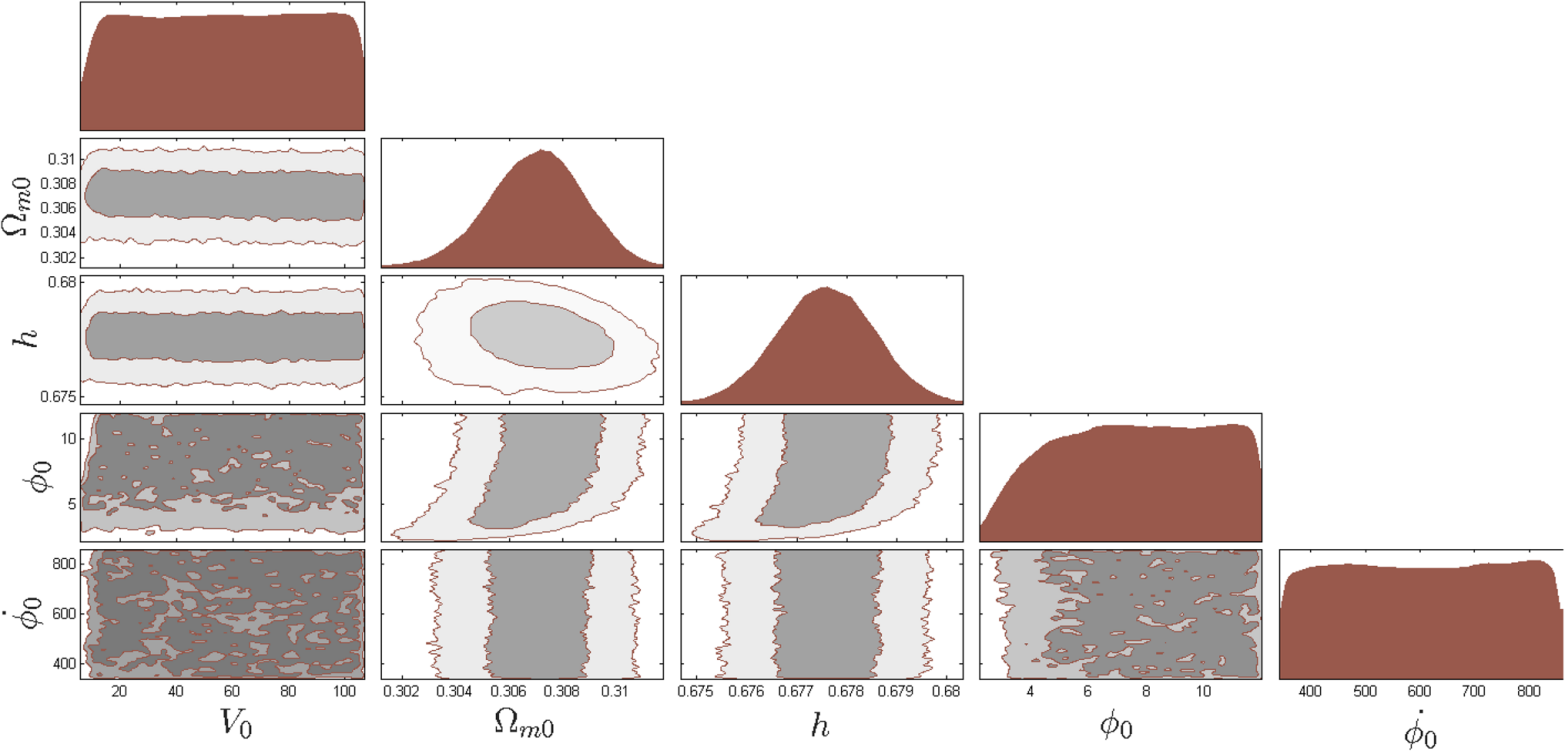
The 2$\sigma$ confidence level contour plots for various pairs of the free parameters ($V_0$, $\varOmega _\mathrm{m0}$, *h*, $\phi _0$, $\dot{\phi _0}$) for which the $\phi$CDM model with the Zlatev–ang–teinhardt potential $V(\phi )=V_0(\exp ({M_\mathrm{pl}/\phi })-1)$ is in the best fit with the $\varLambda$CDM model

**Fig. 5:**
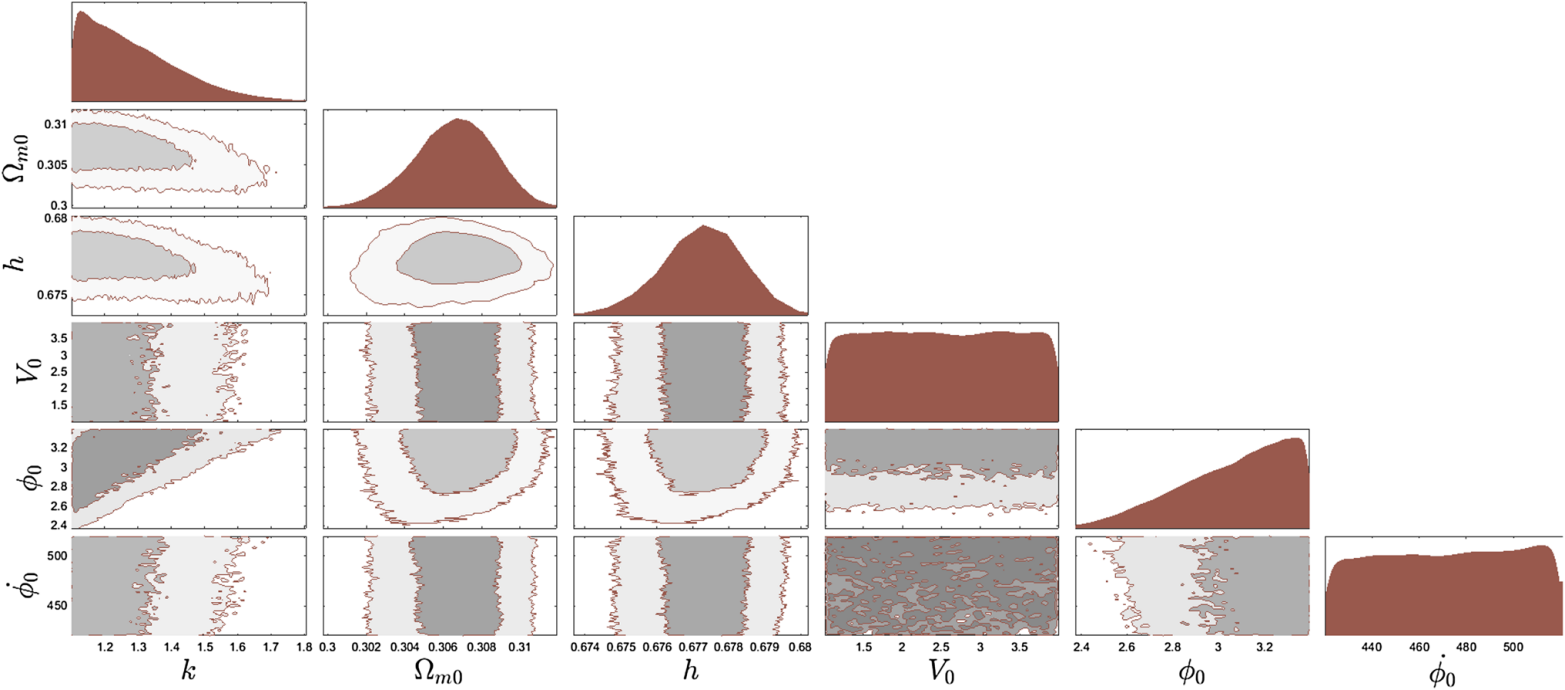
The 2$\sigma$ confidence level contour plots for various pairs of the free parameters (*k*, $\varOmega _\mathrm{m0}$, *h*, $V_0$, $\phi _0$, $\dot{\phi _0}$) for which the $\phi$CDM model with the phantom Pseudo Nambu-Goldstone boson potential $V(\phi )=V_0(1-\cos (\phi /\kappa ))$ is in the best fit with the $\varLambda$CDM model

These results are presented in the Tables 3 and 4. All these numbers are normalized with respect to the fiducial $\varLambda$CDM model.

### The $\phi$CDM potentials vs CPL parametrization

As a additional exercise we looked at how well the CPL parametrization approximates these dark energy models and where each dark energy model is mapped in the CPL parameter space. The CPL-$\varLambda$CDM contours in Figs. 1 and 2 represent 1, 2, and 3$\sigma$ confidence levels for the CPL parametrization derived by fitting the same *H*(*z*), $d_A(z)$, and $f(a)\sigma _8$ data. In order to check how well the CPL parametrization Eq. (8) describes the dark energy models, we find the best-fit effective values of $w_0-w_a$ for a range of the free parameters of each model. These results are presented in Fig. 1 for the quintessence models and in Fig. 2 for the phantom models. For an easy visual representation of this information we pick a parameter with respect to which the best fit $w_0$ and $w_a$ values are most sensitive and plot this range within priors.

In Fig. 1 we show that some of the dark energy models stay very close to the $\varLambda$CDM for a wide range of parameter values within our priors. The range of the EOS parameters for the Ferreira-Joyce, the inverse exponent and the Sugra potentials is very small, it almost coincides with the $\varLambda$CDM model EOS parameter ($w_0=-1, w_a=0$) consequently the likelihood of these model parameters is relatively flat and they can only be distinguished from $\varLambda$CDM model by Occam’s razor type arguments. The Chang–Scherrer, the Urẽna–López–Matos, and the Barreiro potentials can result in up to 3$\sigma$ offsets from $\varLambda$CDM for some parameter values; the Zlatev–Wang–Steinhardt, the Ratra–Peebles, the Albrecht–Skordis, and the Sahni–Wang potentials even extend beyond 3$\sigma$ confidence level. This suggests that a significant fraction of the parameter space can be distinguished based on posterior likelihood. All phantom potentials in Fig. 2, except the quadratic potential, exhibit a similar behaviour. The quadratic potential lies outside the 3$\sigma$ contours of projected DESI constraints. This happens because in this model it is difficult to get a $\varLambda$CDM limit with a natural choice of parameter values and initial conditions.

## Conclusions

We have derived projected constraints on a number of dark energy models by fitting them to a mock *H*(*z*), $d_A(z)$, $f(a)\sigma _8(z)$ data generated in a fiducial $\varLambda$CDM model. When fitting to predicted data one has to choose a fiducial model. In our case this fiducial model was a Planck normalised $\varLambda$CDM. While it is obvious that under this scheme the $\varLambda$CDM can never be inferior to its alternatives, it is not clear a priori how strong the evidence in favour of the $\varLambda$CDM model be. Our main goal was to see whether the various Bayesian criteria would provide sufficient evidence in favour of $\varLambda$CDM as opposed to considered alternatives. Our results seem to suggest that even though all the scalar models have a $\varLambda$CDM limit (which obviously remains a good fit to the data) DESI is capable of providing enough evidence to reject them. Our conclusions come with the caveat that they depend strongly on the adopted assumptions about the priors. The kinds of Bayesian arguments that we employed can be very sensitive to the assumed prior range of model parameters [68]. Our priors were reasonably wide and encompassed all of the parameter space that was in the general $\varLambda$CDM “neighbourhood” and thus compatible with currently available data. Physically motivated restrictions on the parameter space would make the rejection of the alternative models more difficult.

In Figs. 3, 4 and 5 we show examples of the constraints that we obtain for the quintessence Ratra–Peebles, the Zlatev–Wang–Steinhardt potentials and for the phantom Pseudo Nambu–Goldstone potential. Since all models have the $\varLambda$CDM model as their limit, strictly speaking it is impossible to rule them out based on the likelihood arguments alone. Therefore we also used commonly cited model comparison criteria in the Bayesian statistics such as the Bayes factor, the *AIC* and *BIC* information criteria. Computing *AIC* and *BIC* in our setup is straightforward. Since all models have the same maximum likelihood by the construction the *AIC* and the *BIC* become simply functions of the number of the extra parameters. To compute the Bayes factors we integrated the posterior within the bounds given in the Tables 1 and 2. The results of the *AIC*, *BIC*, and Bayes factors for all the dark energy models are summarized in the Tables 3 and 4. These numbers clearly demonstrated that if the $\varLambda$CDM model is the true description of dark energy, the full DESI data will be able to strongly discriminate most scalar field dark energy models currently under consideration. These results however need to be taken with a grain of salt. The evidence values are very sensitive to the prior ranges. We only restricted the prior range based on constraints, by using the phenomenological method developed by us. Further restriction of the parameter ranges could significantly increase the evidence value. The results were derived assuming a fiducial $\varLambda$CDM model and the low value of evidence simply means that the model would be easier to discriminate if $\varLambda$CDM was the true model. The flip side of this is that if instead the dynamic dark energy models were true that would also show up more obviously in the data.

We also explored how the dark energy models are map-ped to the CPL parameter surface. For the models considered in our work this parametrization seems to work reasonably well even for the wide redshift range in a sense that the model predictions are always within one percent of of corresponding CPL predictions.

## Footnotes

1 We assume the flat (suggested by current observations [44]), homogeneous and isotropic universe that is described by the Friedmann–Lemaître–Robertson–Walker (FLRW) spacetime metric, $ds^2 = dt^2 -a^2(t)d\mathbf{x}^2$, where *a*(*t*) is the scale factor (normalized to be unity today $a_0\equiv a(t_0)$), and *t* is the physical time.

2 More precisely, for the freezing quintessence scalar fields the EOS parameter is very close to negative one today, while for the thawing quintessence/phantom scalar fields the EOS parameter deviates slightly from minus one in either direction [37].

3 This potential was investigated earlier by Lucchin and Matarrese [52], as well as by Ratra and Peebles [23]. Although the complete detailed description of the model was given by Ferreira and Joice [27].

4 The critical energy density today $\rho _\mathrm{cr} = {3H_0^2(z)}/({8\pi G})$, where $G=M_\mathrm{pl}^{-2}$ is the Newton constant. The current value of the radiation (relativistic component) and the matter (non-relativistic component) density parameter are designated as $\varOmega _\mathrm{r,0}$ and $\varOmega _\mathrm{m,0}$ respectively; $\varOmega _{\mathrm{\phi }}(z)$ is the dark energy density time dependent parameter. We denote $\varOmega _\mathrm{\phi , 0}\equiv \varOmega _{\mathrm{\phi }}(z=0)$. The condition of the background metric flatness is given, $\varOmega _\mathrm{r,0} + \varOmega _\mathrm{m,0} = 1- \varOmega _\mathrm{\phi , 0}$.

5 Dark energy is sometimes characterized by the EOS parameter only, and the corresponding cosmological model is referred as *w*CDM model [57].

6 For the $\varLambda$CDM model the effective values of the growth index, $\gamma (a)$, have a quasi-constant behavior [59]. For the scalar field models the growth index, $\gamma (a)$, changes in the narrow interval of its values [59, 60].
